# Dual‐Functional Transparent Projection Film with Privacy Protection via Cholesteric Liquid Crystal Polymer Networks

**DOI:** 10.1002/marc.202500080

**Published:** 2025-04-18

**Authors:** Jiahui Dong, Dirk J. Broer, Danqing Liu

**Affiliations:** ^1^ Department of Chemical Engineering and Chemistry Eindhoven University of Technology Groene Loper 3 Eindhoven 5612 AE The Netherlands; ^2^ Institute for Complex Molecular Systems (ICMS) Eindhoven University of Technology Groene Loper 3 Eindhoven 5612 AE The Netherlands

**Keywords:** cholesteric liquid crystal, liquid crystal, privacy protection, transparent display

## Abstract

Transparent projection screens are widely used across various industries for applications that require overlaying digital content onto real‐world environments while maintaining high transparency. However, existing technologies face challenges in balancing transparency, projection quality, and privacy protection. In this work, a novel transparent projection screen is  based on cholesteric liquid crystal (CLC) polymer networks is developed. The CLC networks are designed and fabricated to have a small birefringence to achieve a narrow bandwidth. This allows a selective reflection of designed colors for full‐color projection, while allowing other light to transmit. The CLC films, designed with multi‐layer CLC networks, selectively reflect red, green, and blue light to achieve high‐quality full‐color projection while maintaining transparency. The tri‐layer CLC film demonstrates an average transparency of 70.2% and a color gamut covering 112% of the sRGB standard, ensuring vibrant and accurate color reproduction. To incorporate privacy protection, a hexa‐layer CLC structure is developed, reflecting ≈100% of RGB light and preventing interior light from escaping, while still allowing ambient outdoor light to pass through. This dual functionality highlights the potential of CLC‐based transparent projection films for applications in secure communication, immersive displays, and advanced visual technologies where both transparency and privacy are essential.

## Graphical abstract

This work introduces a novel transparent projection screen using cholesteric liquid crystal (CLC) polymer networks. Multi‐layer CLC films enable high‐quality, full‐color projection with 70.2% transparency and 112% sRGB gamut coverage. A hexa‐layer design adds privacy by blocking interior RGB light. The technology offers promising applications in immersive displays and secure visual communications.

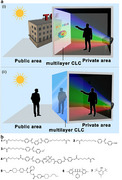

## 1 Introduction

In recent years, smart windows and smart glass technologies have gained considerable attention for their ability to dynamically control light transmission, enhance privacy, and improve energy efficiency in modern buildings.^[^ ^1^, ^2^, ^3^ ^]^ Liquid crystal (LC)‐based materials have emerged as a promising solution for these applications due to their ability to switch between transparent, translucent, and opaque states by manipulating the alignment of their molecules.^[^ ^4^, ^5^, ^6^, ^7^, ^8^, ^9^ ^]^ Building on the principles of LC‐based smart windows, transparent projection screens have emerged as an extension of this technology, where images, videos, or other visual content can be projected onto a transparent surface.^[^ ^10^ ^]^ This allows viewers to see through the display, while overlaying digital content onto the real‐world environments. This innovation has gained widespread adoption across various industries due to its versatile applications. For instance, in the retail sector, transparent screens are commonly used on storefront windows, allowing passersby to view advertisements while simultaneously seeing the products inside the store. In modern corporate environments, where office space is often at a premium, windows or glass walls are increasingly being transformed into transparent presentation screens.

**Figure 1:**
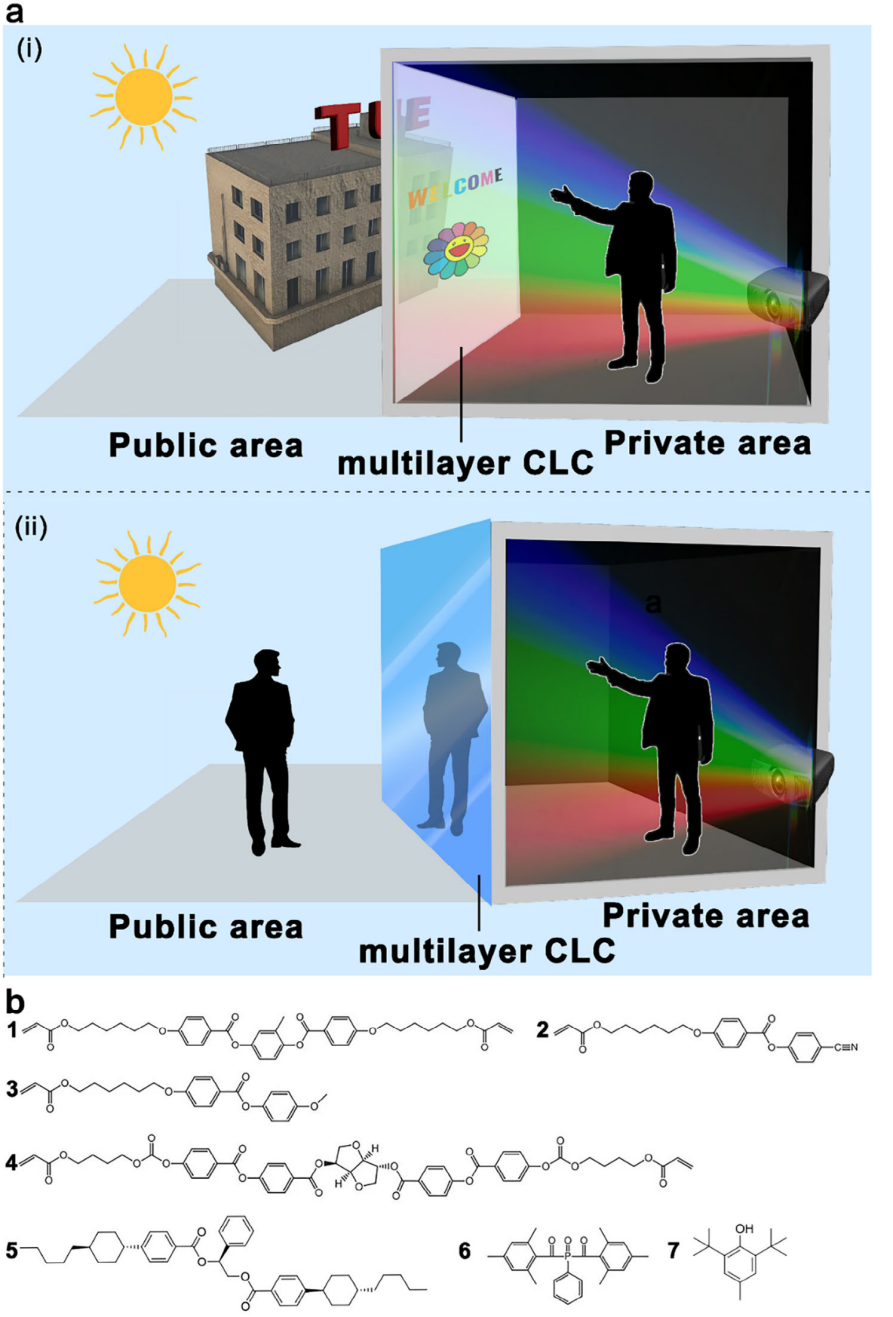
Design concept of dual‐functional multi‐layer CLC polymers for transparent projection film with privacy protection. a) Schematic illustration of the transparent projection function (i) and privacy protection function (ii). b) Material composition of the CLC networks. (**1, 2**s and **3** LC monomers, **4** right‐handed chiral dopant, **5** left‐handed chiral dopant, **6** photoinitiator, **7** polymerization inhibitor).

The current technologies developed for fabricating transparent projection screens face several challenges: i) achieving a balance between high gain and sufficient transparency remains difficult, as seen in methods like microlens arrays with partially reflective coatings^[^ ^11^ ^]^ and nanoparticle‐embedded films,^[^ ^10^, ^12^ ^]^ where improved gain often comes at the cost of reduced transparency; ii) these screens allow visibility from both inside and outside, exposing the projected content or indoor activities to external observers. This increases the risk of unintentional exposure of sensitive or confidential information. Therefore, our aim is to develop a technology that offers dual functionalities: i) transparent projection, allowing individuals inside a room to enjoy both the projected content and the outdoor environment simultaneously (**Figure** **1**a‐i; and ii) integrated privacy protection, preventing individuals outside the room from viewing the interior, effectively safeguarding privacy without compromising the transparency for those inside (Figure 1b‐ii).

For this purpose, we propose the development of a transparent projection screen based on cholesteric liquid crystal (CLC) polymer networks. CLCs are a type of LCs that exhibit a helical molecular structure, enabling them to selectively reflect specific wavelengths of light based on the pitch of the helix.^[^ ^13^, ^14^, ^15^, ^16^ ^]^ To achieve high transparency and projection quality, CLC networks with a small birefringence (*Δn*) were used, which resulted in a narrow reflection bandwidth.^[^ ^17^ ^]^ This narrow bandwidth enhances transparency by allowing non‐reflected light to transmit while selectively reflecting the designed colors for projection. For full‐color projection, the multi‐layer CLC film was engineered to reflect the primary additive colors, red, green, and blue,^[^ ^18^ ^]^ by adjusting the pitch of each CLC layer to target specific wavelengths.^[^ ^19^ ^]^ The screen offers full‐color projection capability with high transparency, allowing users to experience an enhanced visual display while still maintaining a clear view of their surroundings. Additionally, by incorporating both right‐handed and left‐handed CLC layers in the multi‐layer film, a groundbreaking privacy protection function is introduced, preventing outside observers from seeing into the room. This dual functionality greatly enhances the versatility of transparent projection screens, paving the way for new possibilities in secure communication environments and immersive user experiences.

## 2 Results and Discussion

In order to reflect a desired color, a helical structure must be established.^[^ ^20^, ^21^ ^]^ A right‐handed chiral dopant (molecule **4**) was added to the nematic reactive monomers (molecules **1**, **2**, and **3** in Figure 1b) to form the CLC network. The ratio of **1**, **2**, and **3** was carefully optimized to ensure an appropriate operational temperature range for the nematic phase, as well as to provide suitable mechanical properties for the film's functionality. The reflected wavelength can be adjusted by the concentration of the added chiral dopant (Figure S1, Supporting Information). The UV‐VIS spectrum shows that the CLC networks are able to reflect red, green, and blue light, respectively, with 3.6%, 4.6%, and 5.3% molecules **4**.

**Figure 2:**
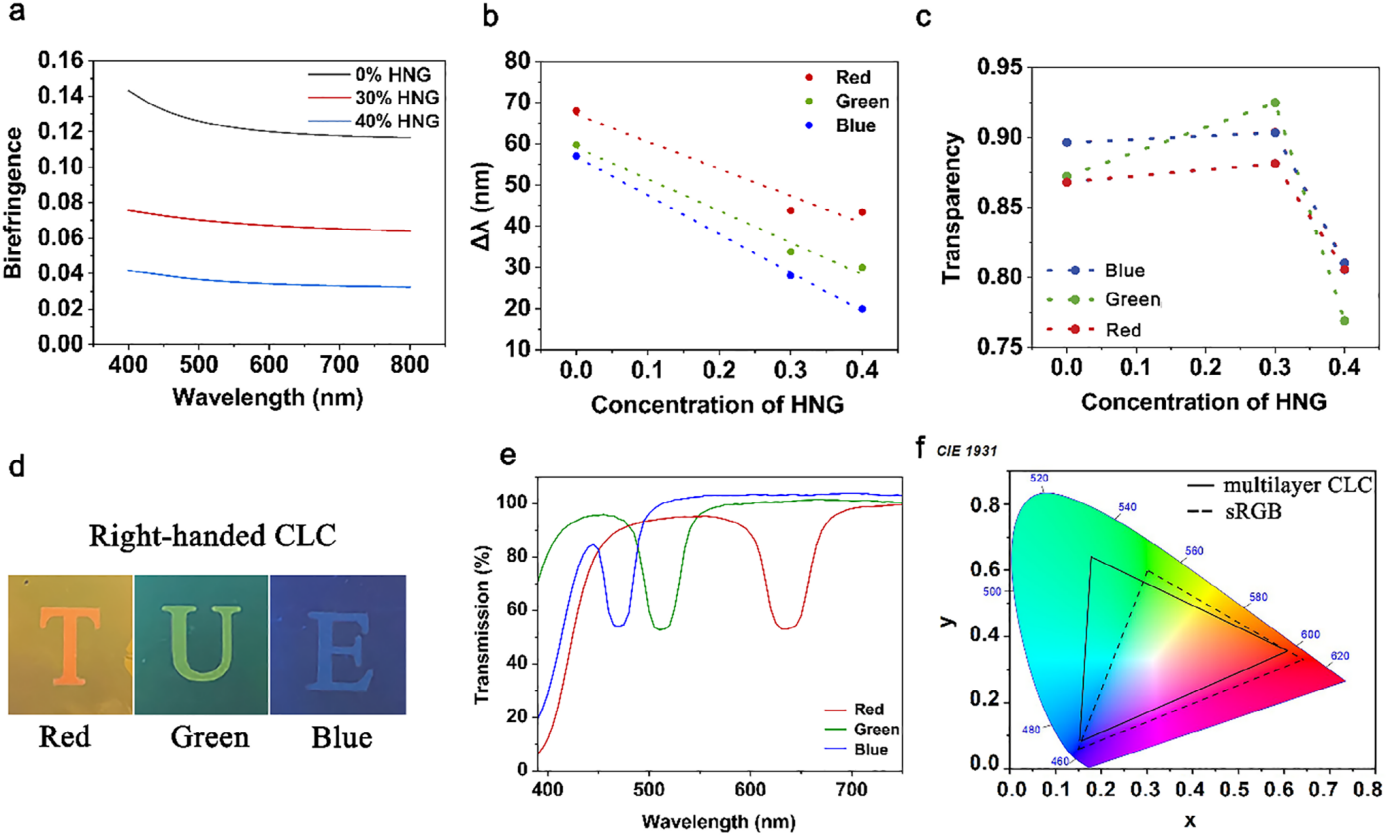
Optical characterization of the fabricated CLC networks. a) Birifringence (Δn) of the LC film with different HNG concentrations within visible wavelength range. b) Bandwidth (Δλ) of the red, green, and blue‐reflection peak with various HNG concentrations. c) The calculated average transparency of the red, green, and blue‐reflective spectra with various HNG concentrations. d) Photographs of the right‐handed single‐layer CLC films, with letters as background, showing their transparency. e) Transmission spectra of the right‐handed single‐layer CLC films. f) The CIE 1931 color gamut shows the color gamut of the right‐handed CLC films, compared with the standard sRGB color gamut.

To achieve a higher transparency and highly saturated color, a narrow reflection (or transmission) band is preferred, which allows more light at other wavelengths to transmit. The bandwidth is determined by the *Δn* of the material by: *Δλ  =  pΔn*, where *Δλ* is the bandwidth, *p* is the helix pitch of the CLC, *Δn* is the birefringence of the CLC.^[^ ^22^, ^23^ ^]^ Therefore, an LC mixture with a small *Δn* is essential to be used for producing a transparent projection film with a narrow bandwidth, and therefore, a higher transparency. The *Δn* of the current LC mixture is measured via ellipsometry (Experimental section), which is 0.12–0.14 in the visible range (**Figure** **2**a). To decrease the *Δn*, we added molecules with *Δn* = 0.04 at 589 nm, as denoted as HNG in the following. As a result, *Δn* of the adjusted mixture is decreased to 0.06‐0.08 with 30% HNG, and to 0.03–0.04 with 40% HNG (Figure 2a).

With the success in decreasing *Δn* of the LC mixture, we further check if this helps to narrow the bandwidth and therefore, to increase the transparency of the CLC. UV–Vis spectrum analysis confirms that the wavelength bands (at all of red, green, and blue wavelengths) have indeed been narrowed with the optimized CLC mixtures (Figure S2, Supporting Information). The bandwidths in these spectra were measured, as shown in Figure 2b. They exhibit a decrease trend with the increase of the HNG concentration. For the peaks at the same central wavelength, the bandwidth decreases ≈35–50% with 30% HNG and ≈36–65% with 40% HNG in the CLC. The narrowest bandwidth we can reach here is 20 nm at wavelength of blue light, which is much lower than the current reported values.^[^ ^24^ ^]^ We calculated the average transparency from the obtained spectra. With the addition of 30% HNG, the transparency indeed increases as expected (Figure 2c). However, it decreases largely when adding 40% HNG, which may be caused by severe phase separation and disruption of the alignment by the non‐reactive molecules in HNG. Therefore, 30% HNG‐containing CLC mixture was further used for producing right‐handed CLC and their corresponding characterization. Furthermore, the optimized transparency can reach about or higher than 90%, which would provide a high visibility of the behind objects. This is proved by Figure 2d. The “T”, “U”, and “E” logo remain highly visible underneath CLC films, and at the same time, CLC films reflect red, green, and blue colors, respectively (Figure 2d). Good visibility was observed both in direct contact with the logo and from a distance over 1 meter (Figure S3, Supporting Information). The red‐reflective film is centralized at the wavelength of 630 nm, and green one at 510 nm, and blue one at 470 nm (Figure 2e). All of the red‐, green‐and blue‐reflective CLC polymer films reflect almost 50% unpolarized light, indicating their good alignment.^[^ ^25^ ^]^

Besides transparency, the color gamut is another critical parameter for a transparent projection film. A wider color gamut allows more precise color production,^[^ ^26^ ^]^ resulting in projected contents with greater display fidelity and visual quality. A narrow bandwidth of the spectrum often leads to a wider color gamut (Figure S4, Supporting Information). We plotted the color gamut of red, green, and blue‐reflective CLC polymers in CIE 1931 coordinates together with sRGB standard color space. The color gamut of CLC film was measured to cover 112% of the sRGB standard color space (Figure 2f). Our fabricated film exhibits a significantly broader color gamut compared to other reported films, which have color gamut of 21%, 46%, or 76%.^[^ ^24^ ^]^ The wide color gamut suggests that the CLC film not only meets but exceeds the requirements for high‐quality visual displays, providing enhanced color vibrancy and accuracy.

### 2.1 Full‐Color Transparent Projection Function

**Figure 3:**
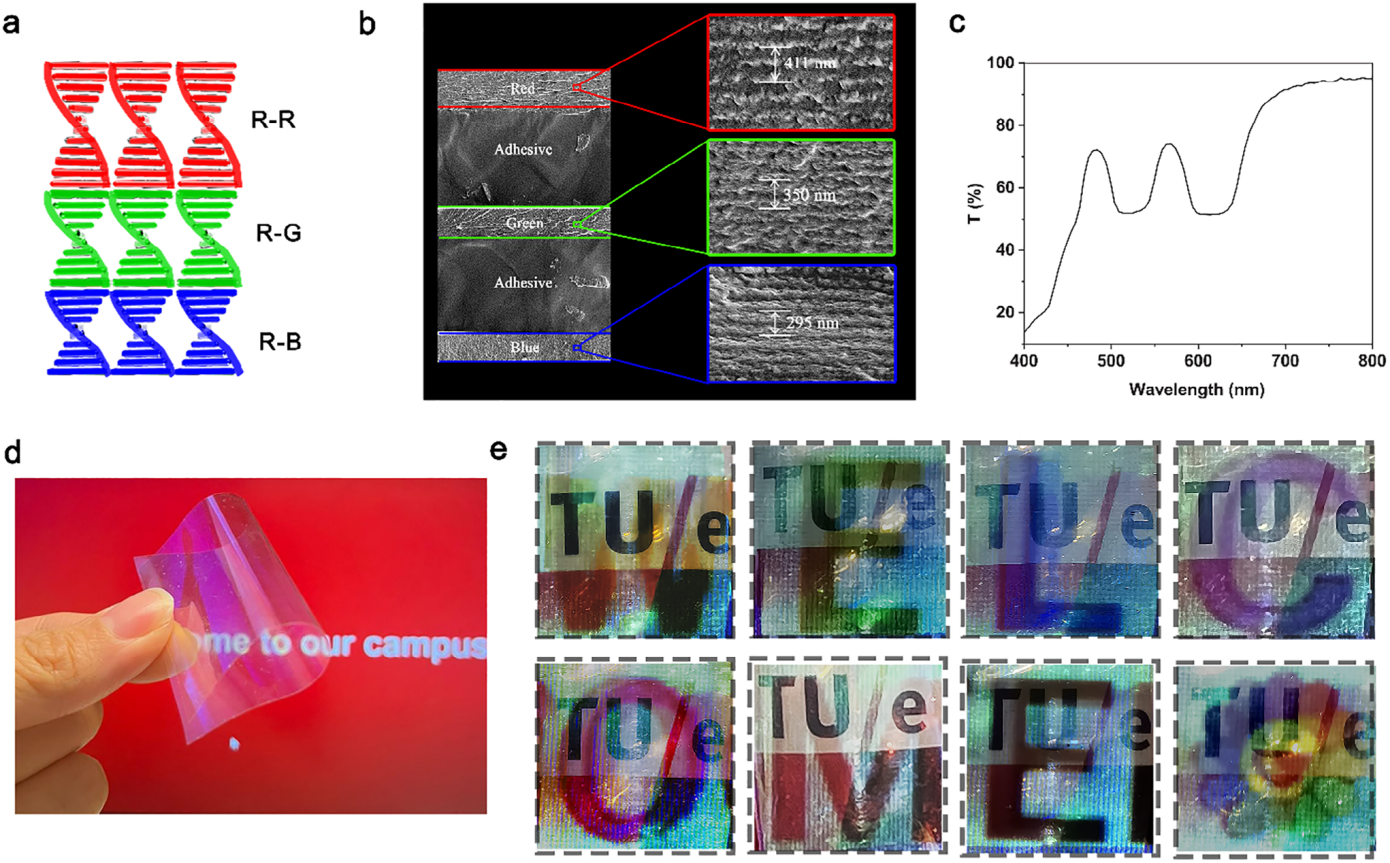
The structure and characterization of the tri‐layer CLC film, as well as its demonstration for transparent projection function. a) Schematic illustration for the design of the tri‐layer transparent projection film. b) Cross‐sectional SEM images of the stacked CLC film and the measured pitches for each layer. c) Transmission spectra. d) Demonstration of the fabricated CLC polymer film, showing its free‐standing and flexible features. e) Demonstration of the CLC film shows full‐color projection with the logo clearly visible in the background.

To achieve a full‐color projection, the film should be capable of reflecting the primary additive colors: red, green, and blue (RGB). Therefore, a multilayer of CLC film is designed to be structured into at least three distinct layers reflecting red, green, and blue, respectively, which are denoted as “R‐R”, “R‐G”, and “R‐B” (**Figure** **3**a). We stacked “R‐R”, “R‐G” and “R‐B” together with an adhesive in between. The cross‐sectional view of the stacked tri‐layer CLC structure was observed under scanning electron microscope (SEM) (Figure 3b), where the pitches of each layer are clearly visible in the image. The measured pitches were 411, 350, and 295 nm for the red, green, and blue‐reflective layers, respectively. These pitch values are consistent with the expected reflection wavelengths for each color.

The UV‐VIS spectrum of the fabricated tri‐layer CLC structure exhibited distinct reflection peaks corresponding to the red, green, and blue regions of the visible spectrum (Figure 3c). The tri‐layer CLC structure selectively reflects ≈50% of the incident red and green light. In the blue region, a much deeper reflection peak was observed. This can be attributed to the overlap between the reflection peak of the blue light and the absorption band of the photoinitiator (molecule **6**) used during the fabrication process. Despite these reflective properties, the overall transparency of the tri‐layer CLC film remained high, with an average transparency calculated to be 70%. Figure 3d demonstrates that the fabricated CLC polymer film is free‐standing and flexible. This free‐standing film can be attached to any transparent surface. Additionally, it can be easily bent while maintaining its transparency, making it suitable for not only flat, but also curved surfaces, such as curved windows or windshields.

To demonstrate the feasibility of the full‐color transparent projection film using multi‐layer CLC, we used a standard projector to project various contents onto the surface of the tri‐layer CLC film, with an object with “TUE” logo behind the film (Figure 3e). When letters of different colors were projected onto the film, the CLC film accurately displayed the colors with high fidelity and the shape of the letter with clear edges. Simultaneously, the TUE logo remained clearly visible through the film. A colorful pattern, containing a variety of rainbow colors, was also projected onto the film. The film successfully rendered the pattern with precise color reproduction, with the “TUE” logo being visible (Figure 3e). These demonstrations underscore the effectiveness of the CLC‐based transparent projection film in achieving high‐quality, full‐color displays while maintaining the essential transparency of the surface.

### 2.2 Privacy Protection Function

Next, to possess an additional privacy protection function for the transparent projection film, the CLC films need to be engineered to block the transmission of projected or interior light to the outside, while allowing exterior ambient light to pass through. To achieve this goal, the design of the multi‐layer film incorporates right‐handed and left‐handed CLC with the same pitch in the same structure. When using the RGB light source for the private space, this structure ensures that at most 100% of the red, green, or blue light or their combination is reflected back into interior, which prevents any interior light from escaping through the film, therefore, enhancing the privacy function of the CLC films. Simultaneously, the film allows ambient light that contains a broader spectrum of wavelengths to partially pass through to the interior, preserving the indoor viewer's ability to see the external environment.

To produce such privacy CLC film, in addition to right‐handed CLC networks, left‐handed CLC networks were obtained by mixing the same monomers (molecules **1**, **2,** and **3**) with a left‐handed chiral dopant (molecule **5**). The optimization of the left‐handed CLC networks for transparency is presented in Figure S5a (Supporting Information). Unlike the right‐handed CLC, the addition of HNG did not result in an increase in transparency for the left‐handed CLC networks, despite narrowing their reflection bandwidth. This reduced transparency is attributed to scattering losses, particularly at shorter wavelengths,^[^ ^25^, ^27^ ^]^ due to severe phase separation of non‐reactive molecules within the left‐handed chiral dopant and HNG.^[^ ^28^ ^]^ As a result, the left‐handed CLC networks without any HNG was selected for further characterization and demonstration. The spectra of the red, green, and blue‐reflective left‐handed CLC networks are presented in Figure S5b (Supporting Information).

**Figure 4:**
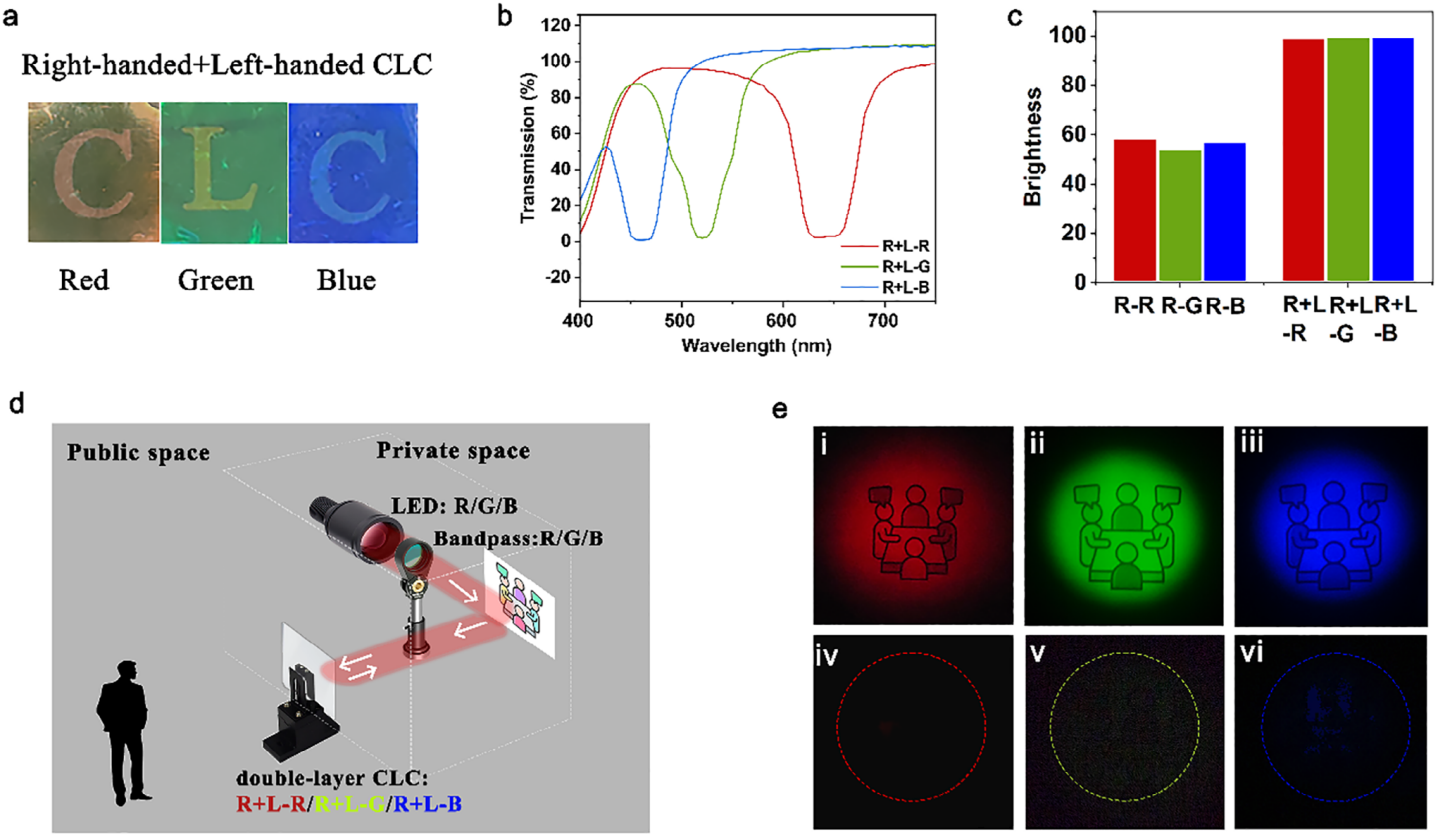
The structure and characterization of the combination of right‐handed and left‐handed CLC film, as well as its demonstration for privacy protection function. a) The right‐handed and left‐handed (with the same pitch) double‐layer CLC films, with letters as background, showing their transparency. b) Transmission spectra of the right‐handed and left‐handed (with the same pitch) double‐layer CLC films. c) The brightness of right‐handed single‐layer CLC films and right‐handed and left‐handed (with the same pitch) double‐layer CLC films. d) Schematic illustration of the experimental setup, performed in dark, for evaluating the privacy protection function of the double‐layer CLC film. LED lights combined with bandpass filters were used as the light source for the private space. e) Photographs of the logo in the private space, taken from the public space, without (i, ii, and iii) and with (iv, v, and vi) the double‐layer CLC film inserted between the private and public spaces.

Then, we stacked the CLC networks with opposite handedness together to form a double layer CLC, as shown in **Figure** **4**a. The letters “C”, “L”, and “C” underneath the films are still clearly visible. The double‐layer film that reflect red, green, and blue, respectively, which are denoted as “R + L‐R”, “R+L‐G”, “R + L‐B”, respectively. All of the double‐layer films reach close to 0% transmission (i.e., 100% reflection) at the central wavelengths (Figure 4b). The color gamut of a film that composed of “R + L‐R”, “R + L‐G” and “R + L‐B” covers less color space compared with the one with only right‐handed CLC networks (Figure S6, Supporting Information).

The double‐layer film (i.e, R + L‐R, R + L‐G, or R + L‐B) is expected to have a higher brightness than the single layer (i.e, R‐R, R‐G, or R‐B). To quantify this, we transformed the colors in RGB color space into hue saturation brightness (HSB) color space and obtained the values for brightness (Figure 4c). The brightnesses of the single‐layer films are ≈50–60, while those of the double‐layer films are ≈100.

To further investigate the privacy protection function of the CLC film, we evaluate the double‐layer CLC (i.e, R + L‐R or R + L‐G, R + L‐B) by making a demonstration in a dark environment (Figure 4d). An LED with narrow bandpass filter was used to produce light within specific wavelength range, precisely matching the CLC film's reflection band to ensure complete light reflection. The light was directed at a logo, which represents an object located within a private area. A camera positioned outside this private area, with a film holder placed between the camera and the logo. Initially, without the CLC film in place, the camera could clearly capture the illuminated logo, simulating a scenario where the private activity would be visible to an external observer (Figure 4e‐i–iii)). However, when the CLC film, R + L‐R, R + L‐G, or R + L‐B was inserted into the setup, all the light (the produced red, green, or blue light) reflected by the logo was reflected back into the private area by the double‐layer CLC structure. As a result, the camera, representing an external observer, could not detect any light from the private area and recorded only darkness (Figure 4e‐iv–vi).

### 2.3 Full Color Transparent Projection with Additive Privacy Protection

**Figure 5:**
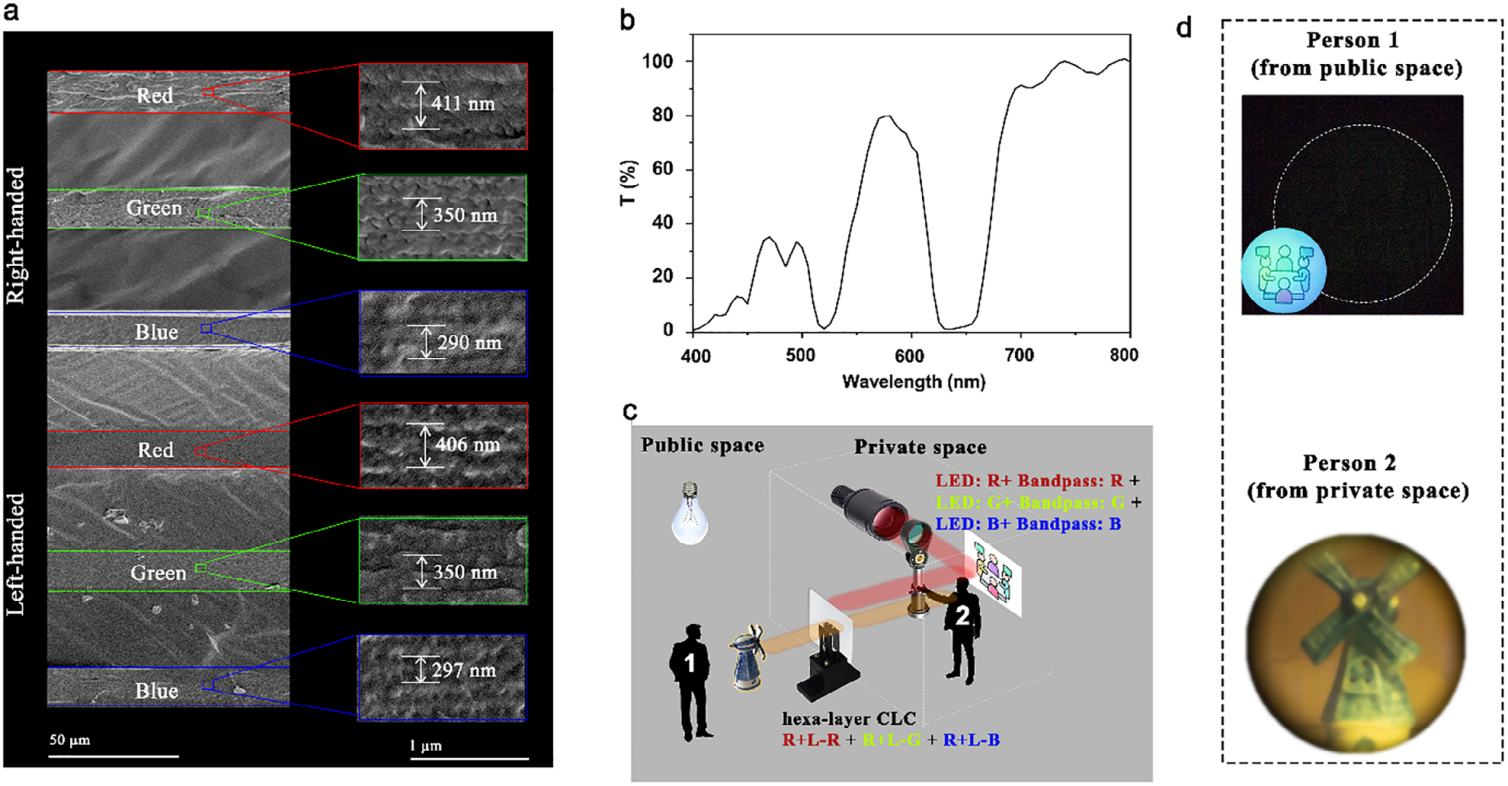
Structure and characterization of the hexa‐layer CLC film, along with its demonstration for privacy protection function. a) Cross‐sectional SEM images of the stacked CLC film, showing the measured pitches for each layer. b) Transmission spectra of the hexa‐layer film. c) Schematic illustration of the experimental setup, performed in the dark, for evaluating the privacy protection function of the hexa‐layer CLC film. LED lights combined with bandpass filters were used as the light source for the private space, while a fluorescent light was used in the public space to simulate a real‐world scenario. d) The view from “person 1″, representing the external observer, with the hexa‐layer CLC film inserted (The inset in person 1′s view shows the scenario without the CLC film), and the view from “person 2″, representing the people located in the private space.

To achieve both full‐color transparent projection and privacy protection on one film, we stacked three sets of right‐ and left‐handed CLC networks: “R + L‐R” for red light, “R+L‐G” for green light, and “R + L‐B” for blue light. The SEM image of the cross‐section of the stacked hexa‐layer CLC structure reveals tightly packed layers with adhesive applied between each layer to ensure structural integrity (**Figure** **5**a). The measured pitches for each layer align with the expected central wavelengths of each CLC film. The optical spectrum of the hexa‐layer CLC film shows that it reflects ≈100% of the red, green, and blue wavelengths (Figure 5b). Importantly, light at other wavelengths can still pass through the film, maintaining partial transparency. The calculated average transparency of the hexa‐layer CLC film is 49%, demonstrating that while it provides robust privacy protection by reflecting the key RGB light, it still allows sufficient light transmission to retain a degree of transparency.

In assessing the privacy protection function of the hexa‐layer CLC film, we employed a combination of blue, green, and red LEDs, each paired with a corresponding bandpass filter to ensure precise wavelength control (Figure 5c). These three light beams were simultaneously directed at a logo situated in the private space, simulating an illuminated object within a confidential area (Figure 5d). An ambient fluorescent light was used in the public space, consisting of wavelengths outside the reflection range of the CLC layers. With the hexa‐layer film in place, all three primary colors were effectively reflected back into the private area. Therefore, an external observer (designated as “person 1” (Figure 5c)) was unable to see the illuminated logo (Figure 5d). In contrast, “person 2”, located inside the private space (Figure 5c), could still observe the public space through the CLC film (Figure 5d). This demonstrates that the hexa‐layer CLC film can successfully provide privacy protection by preventing external observation while maintaining visibility for those inside the private space.

## 3 Conclusion

In conclusion, our CLC polymer network‐based films present a significant advancement in transparent projection technology. It offers dual functionality of high‐quality projection and privacy protection. By utilizing red, green, and blue (RGB)‐reflective CLC networks with low birefringence, we achieved full‐color projection with improved transparency. The tri‐layer CLC film demonstrated 70% transparency and a color gamut of 112% sRGB, while the hexa‐layer configuration ensured privacy by enabling indoor viewing while blocking external visibility.

The successful development of this CLC‐based transparent projection film could accelerate augmented reality (AR) technology across diverse fields, smart healthcare for precise surgical procedures, smart homes for immersive experiences, confidential office environments for secure presentations, remote communication for seamless spatial interaction, the automotive industry for enhanced head‐up displays, and museums for immersive cultural engagement. With easy application to any flat or curved glass surface and no need for complex setup, this film makes advanced digital overlays accessible and integrates digital interaction into everyday life.

## 4 Experimental Section

### Materials

Figure 1c shows the components for LC monomer mixtures. LC monomers **1–3** were obtained from Merck UK. Molecule 4 (Paliocolor LC756, right‐handed chiral dopant) was obtained from BASF (Schweiz AG), and molecule 5 (S1011, left‐handed chiral dopant) was purchased from Merck Germany. Molecule **6** (Irgacure 819) was purchased from Ciba Specialty Chemicals. Molecule **7** (Butylated hydroxytoluene) was obtained from Sigma–Aldrich. The molecule mixture with Δn = 0.039 at 589 nm was purchased from Jiangsu Hecheng Display Technology Co., Ltd (HCCH). Typically, the ratio of **1**, **2,** and **3** is 1:3:2. The ratio of molecule **4** or **5** varied, depending on the desired reflection wavelength. 1% molecule **6** and 0.5% molecule **7** were used in the mixtures. All the LC mixtures were made by dissolving them in dichloromethane and then evaporating the solvent at 50 °C.

### Sample Preparation

CLC films were fabricated by photopolymerizing the LC monomer mixture in a cell at 45 °C. The cells were formed by gluing two glass substrates with a gap of 10 µm as confined by spherical beads (Sekisui Chemical Co., Ltd.).  For a single layer of CLC film, one of the glass substrates was precoated with polyimide layer (AL 1254, JSR Micro, Belgium) for planar alignment. The other substrate was precoated with 3‐(trimethoxysilyl) propyl methacrylate (Sigma–Aldrich) to anchor the CLC coating. The precoated glass substrates were briefly sheared along the rubbing direction to promote planar alignment of the chiral‐nematic LC mixture. The LC monomer mixture was filled in cells at its isotropic phase and then cooled down to nematic phase. Next, the mixture was polymerized by a UV lamp (Omnicure EXFO S2000). After photopolymerization, the glass substrate with a polyimide alignment layer was removed.

For the fabrication of stacked multiple‐layer of CLC, the bottom single CLC layer (red‐reflective CLC film in this work) of the structure was first fabricated by using the same procedure as used for single layer of CLC film as mentioned above. For the upper stacked CLC layer, polyvinyl alcohol (PVA) precoated glass substrates were used for the cells, because both side of PVA coated‐glass of a cell can be easily removed by being immersed in water. A polyethylene terephthalate (PET) adhesive was used between CLC layers to maintain the structural integrity.

### Characterization

The birefringence of the LC network was determined using a spectroscopic ellipsometer (EP4, Accurion GmbH, Germany). Monomer mixtures without chiral components were prepared for ellipsometry measurement. The mixtures were filled into a cell with the gap of 1–2 µm with a homeotropic alignment layer on the glass substrates. Then, one of the glass substrates was removed and the coating samples were ready for the measurements. The measured wavelength spans from 400 to 800 nm. The measured data was modeled with Cauchy/ Cauchy‐Urbach model with the software EP4Model.

The thickness of the CLC films was measured by a profilometer (Dektak XT, Bruker Corporation, USA). The transmission of the CLC single layer or multi‐layer film was measured by a spectrophotometer (PerkinElmer LAMBDA 750 UV/vis/NIR spectrophotometer). Color gamut of the CLC films was generated from the plug‐in App, Chromaticity Diagram, in the Origin 2023b software. The cross‐sectional microstructure of the CLC multi‐layer film was observed with a scanning electron microscope (FEI SEM Quanta 3D FEG). The helix pitches of the CLC films were determined using ImageJ software.

A projector (Ace Ki, Yaber, Prolinx GmbH) was used for the demonstration of transparent projection CLC film. For demonstrating its privacy protection function, LED lamps (M625L4, M530L4, M455L4, Thorlabs, USA) with bandpass (Thorlabs, USA) were used as light source for private space.

## Conflict of Interest

The authors declare no conflict of interest.

## Supporting information



Supporting Information

## Data Availability

Research data are not shared.
