# Supplementary material for: Dual‐Functional Transparent Projection Film with Privacy Protection via Cholesteric Liquid Crystal Polymer Networks
Source: Macromol Rapid Commun. 2025 Apr 18;46(15):2500080. doi: 10.1002/marc.202500080 (PMC12344476; doi:10.1002/marc.202500080)
Supplement: Supplementary file 1 — Supporting Information [file MARC-46-2500080-s001.docx]

Supporting information for

**Transparent projection film with privacy function using cholesteric liquid crystal**

Jiahui Dong, Dirk J. Broer, Danqing Liu

J. Dong, Prof. D. J. Broer, Prof. D. Liu

Department of Chemical Engineering and Chemistry, Eindhoven University of Technology, Groene Loper 3, 5612 AE Eindhoven, the Netherlands

J. Dong, Prof. D. J. Broer, Prof. D. Liu Institute for Complex Molecular Systems (ICMS), Eindhoven University of Technology, Groene Loper 3, 5612 AE Eindhoven, the Netherlands

Email: D.Liu1@tue.nl

**This PDF file includes:**

Supplementary Text

Figure S1 to Figure S5

**Figure S1** shows the UV-VIS spectra of CLC films with different concentrations of right-handed chiral dopant. Liquid crystal mixtures were prepared with varying concentrations of chiral dopants, ranging from 2.8% to 5.3%, and their transmission spectra were measured using UV-VIS spectroscopy. The results demonstrate that increasing the concentration of the chiral dopant shifts the reflected wavelength toward shorter wavelengths, allowing precise tuning of the reflection band. This enables the selection of optimal chiral dopant concentrations to obtain red, green, and blue-reflective CLC formulations for full-color projection applications.


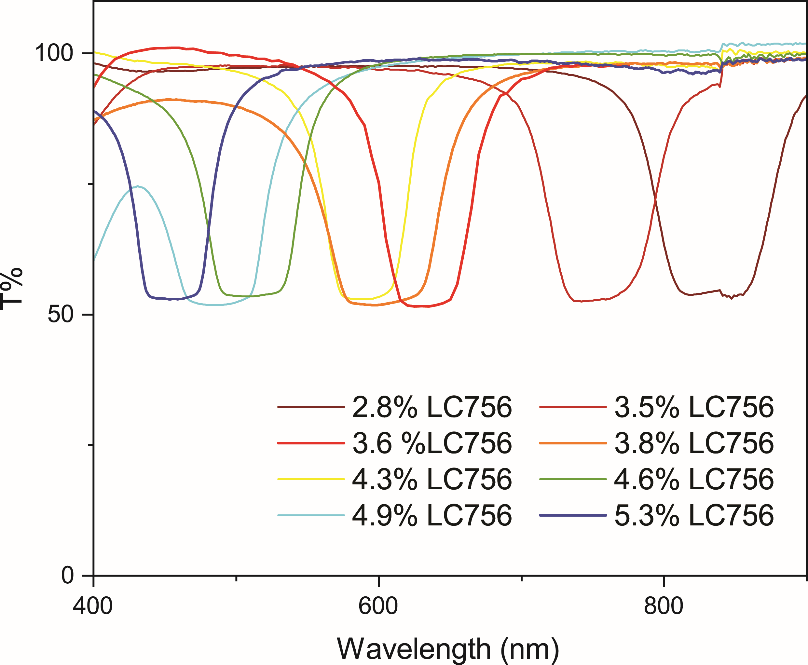


**Figure S1 Transmission spectra of the right-handed CLC with different concentrations of LC756**

**Figure S2** illustrates the effect of adding 30% and 40% HNG into the liquid crystal mixtures on the bandwidth of the transmission peaks. We tuned the concentration of the chiral dopant, LC756, to ensure that the transmission peaks for red, green, and blue were centered at the same wavelengths. The UV-VIS spectrum analysis confirms that the bandwidth of the transmission peaks narrows as the HNG concentration increases. However, the data also reveal that with higher HNG concentrations, scattering losses become more pronounced, particularly at shorter wavelengths. This increase in scattering is likely due to more severe phase separation of HNG from the CLC networks.


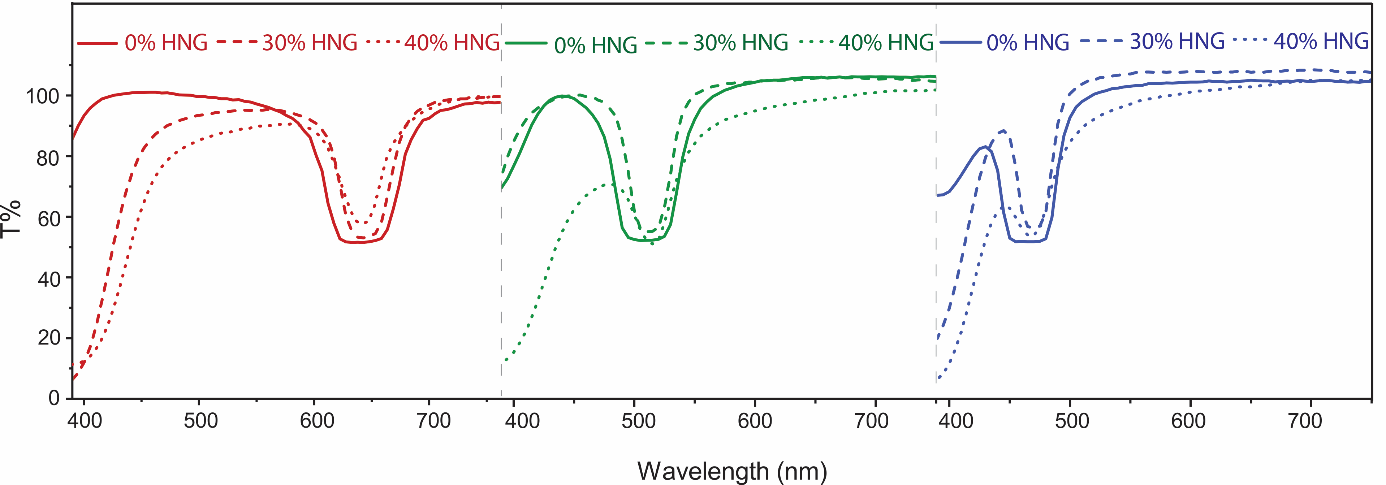


**Figure S2 Transmission spectra of the right-handed CLC showing the narrowing of the bandwidth with an increase of HNG concentrations**

**Figure S3** illustrates the effect of narrowing the bandwidth on the color gamut of the CLC film. The color gamut of the CLC film is shown for both 0% and 30% HNG, with the standard sRGB color space included for comparison. The addition of 30% HNG into the original CLC mixture significantly expands the color gamut, increasing the coverage from 94% to 110% of the sRGB standard. This improvement demonstrates that a narrower bandwidth leads to a wider color gamut, enhancing the film's ability to reproduce vibrant and accurate colors for full-color projection applications.


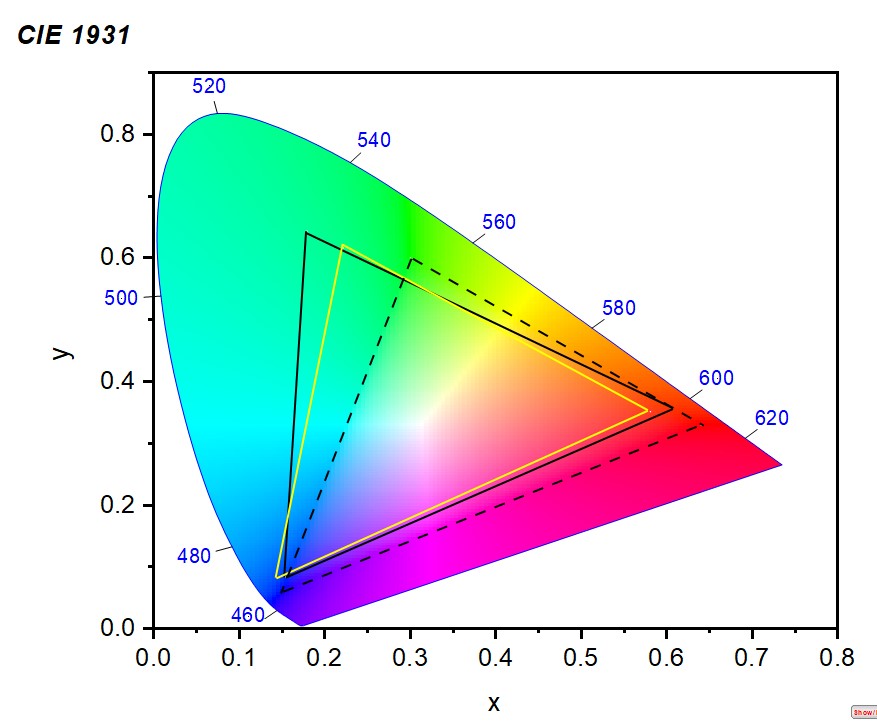


**30% HNG**

**sRGB**

**0% HNG**

**Figure S3 Color gamut of CLC films without and with 30% HNG**

**Figure S4** presents the optimization of the left-handed CLC networks for transparency. **Figure S4a** shows the effect of adding HNG to the red-reflective CLC film, demonstrating that while the bandwidth was narrowed with the addition of HNG, the transparency did not improve due to increased scattering losses at shorter wavelengths. As a result, left-handed CLC films without HNG were selected for further investigation. By tuning the concentration of the left-handed chiral dopant (S1011), we successfully fabricated red, green, and blue-reflective left-handed CLC films (**Figure S4b**) for subsequent experiments.


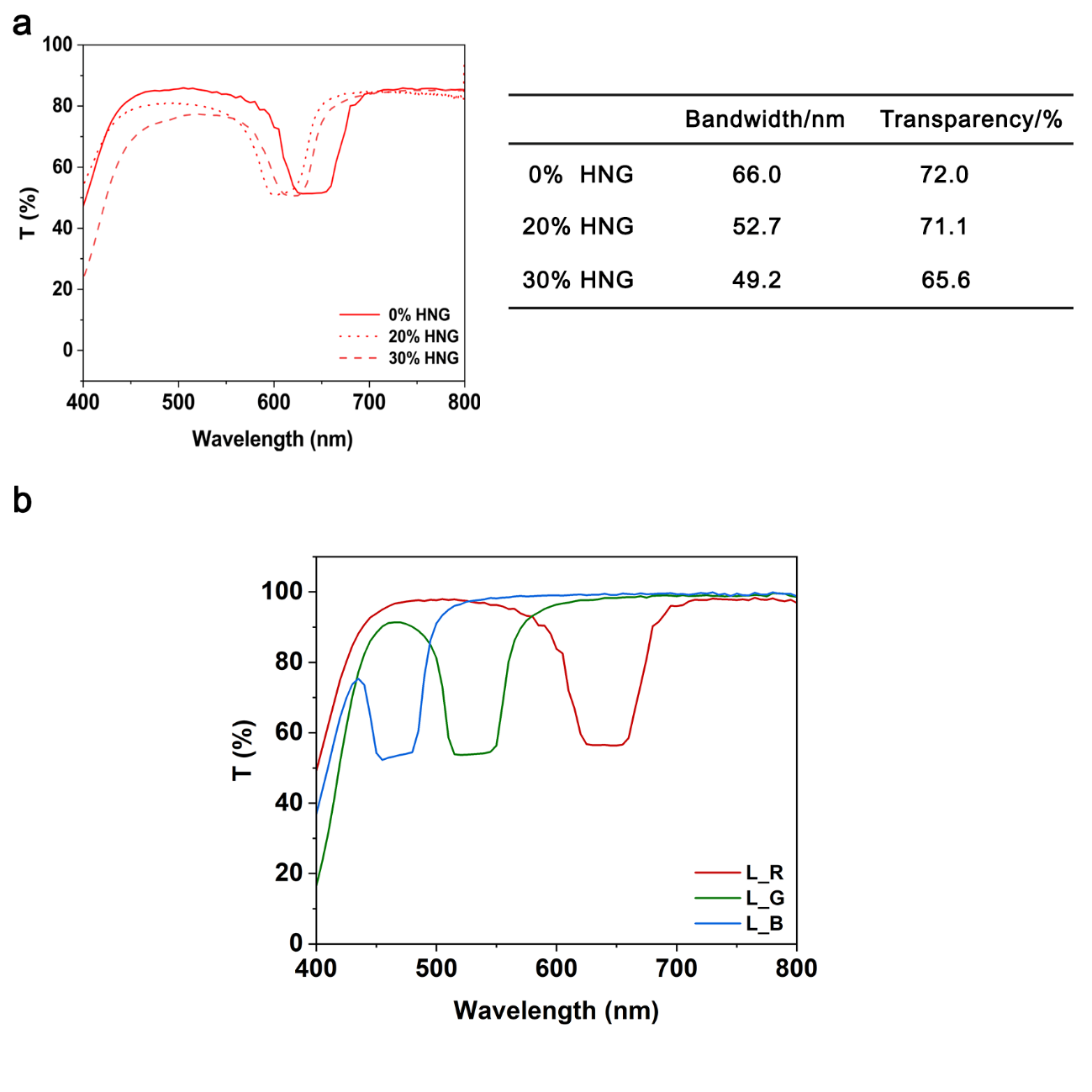


**Figure S4 Transmission spectra of the left-handed CLC. (a) Spectra of the CLC films with different HNG concentrations and their corresponding bandwidth and transparency value. (b) The optimized left-handed CLC films reflecting red, green and blue, respectively.**

**Figure S5** shows the color gamut of the multi-layer CLC film composed of “R+L-R,” “R+L-G,” and “R+L-B” layers. The color space covered by this film is smaller compared to that of a single CLC film. This reduction in color space is attributed to the wider bandwidth of the "R+L" films, which results in less pure color reproduction. The broader reflection bands cause overlapping in the color spectra, leading to a diminished ability to accurately reproduce vibrant and distinct colors.


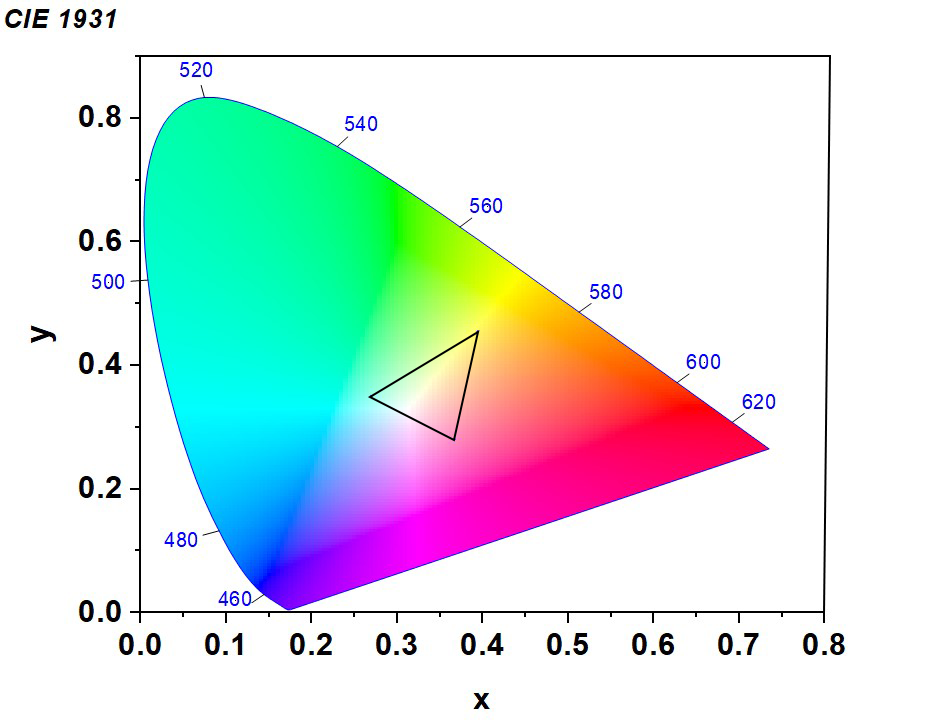


**Figure S5 Color gamut of the multi-layer CLC film incorporating both right-handed and left-handed CLC networks**
